# Potent Activity of Hybrid Arthropod Antimicrobial Peptides Linked by Glycine Spacers

**DOI:** 10.3390/ijms22168919

**Published:** 2021-08-18

**Authors:** Miray Tonk, James J. Valdés, Alejandro Cabezas-Cruz, Andreas Vilcinskas

**Affiliations:** 1 Institute for Insect Biotechnology, Justus Liebig University of Giessen, Heinrich-Buff-Ring 26-32, 35392 Giessen, Germany; miray.tonk@agrar.uni-giessen.de; 2 LOEWE Centre for Translational Biodiversity Genomics (LOEWE-TBG), Senckenberganlage 25, 60325 Frankfurt, Germany; 3 Institute of Parasitology, Biology Centre, Czech Academy of Sciences, Branišovská 1160/31, 37005 České Budějovice, Czech Republic; valdjj@gmail.com; 4 Department of Virology, Veterinary Research Institute, Hudcova 70, 62100 Brno, Czech Republic; 5 Faculty of Science, University of South Bohemia, Branišovská 1160/31, 37005 České Budějovice, Czech Republic; 6 Anses, INRAE, Ecole Nationale Vétérinaire d’Alfort, UMR BIPAR, Laboratoire de Santé Animale, F-94700 Maisons-Alfort, France; alejandro.cabezas@vet-alfort.fr; 7 Fraunhofer Institute for Molecular Biology and Applied Ecology, Branch of Bioresources, Ohlebersweg 12, 35392 Giessen, Germany

**Keywords:** insect, scorpion, antimicrobial peptide, hybrid peptide, glycine spacer, *Escherichia coli*

## Abstract

Arthropod antimicrobial peptides (AMPs) offer a promising source of new leads to address the declining number of novel antibiotics and the increasing prevalence of multidrug-resistant bacterial pathogens. AMPs with potent activity against Gram-negative bacteria and distinct modes of action have been identified in insects and scorpions, allowing the discovery of AMP combinations with additive and/or synergistic effects. Here, we tested the synergistic activity of two AMPs, from the dung beetle *Copris tripartitus* (CopA3) and the scorpion *Heterometrus petersii* (Hp1090), against two strains of *Escherichia coli*. We also tested the antibacterial activity of two hybrid peptides generated by joining CopA3 and Hp1090 with linkers comprising two (InSco2) or six (InSco6) glycine residues. We found that CopA3 and Hp1090 acted synergistically against both bacterial strains, and the hybrid peptide InSco2 showed more potent bactericidal activity than the parental AMPs or InSco6. Molecular dynamics simulations revealed that the short linker stabilizes an N-terminal 3_10_-helix in the hybrid peptide InSco2. This secondary structure forms from a coil region that interacts with phosphatidylethanolamine in the membrane bilayer model. The highest concentration of the hybrid peptides used in this study was associated with stronger hemolytic activity than equivalent concentrations of the parental AMPs. As observed for CopA3, the increasing concentration of InSco2 was also cytotoxic to BHK-21 cells. We conclude that AMP hybrids linked by glycine spacers display potent antibacterial activity and that the cytotoxic activity can be modulated by adjusting the nature of the linker peptide, thus offering a strategy to produce hybrid peptides as safe replacements or adjuncts for conventional antibiotic therapy.

## 1. Introduction

The increasing prevalence of multidrug-resistant (MDR) bacteria and the lack of novel antibiotics in the development pipeline threaten healthcare systems worldwide and have prompted the search for antimicrobial candidates, particularly those with new mechanisms of action against Gram-negative bacteria [1,2]. Antimicrobial peptides (AMPs) are especially promising due to their potent antimicrobial activity and their ability to neutralize toxins [3]. These membrane-active peptides, 10–50 amino acids in length, display activity against bacteria, fungi, viruses and parasites, and are key components of the vertebrate and invertebrate innate immune system [4]. The non-ribosomal antibiotic peptides produced by bacteria and fungi differ from the ribosomal peptides found in higher eukaryotes, and the term AMP usually refers specifically to these latter molecules. AMPs can be assigned to four categories according to their structure and function [5]: linear α-helical peptides, β-sheet peptides, peptides that contain unusually high numbers of specific amino acid residues such as proline or glycine, and mixed α-helix/β-sheet peptides [5]. Where secondary structures and disulfide bridges are present, these are often necessary for AMP activity [6,7].

Insects produce a repertoire of AMPs larger than any other taxonomic group, and a comparative analysis of available genomes and transcriptomes showed that the number of individual AMPs varies considerably from species to species [8,9]. For example, the invasive harlequin ladybird *Harmonia axyridis* is known to produce more than 50 AMPs [8], whereas the pea aphid *Acyrthosiphon pisum* does not produce any known AMPs [9]. This difference in AMP distribution reflects the evolutionary plasticity of insect immunity [8,9]. For example, the harlequin ladybird frequently encounters new habitats with novel pathogens as part of its invasive lifestyle [8], whereas the pea aphid feeds on sterile plant sap and occupies a defined niche. The dependency of pea aphids on bacterial symbionts that provide essential amino acids to compensate for the unbalanced diet has resulted in the loss of AMP genes [9].

The activity of many arthropod AMPs has been characterized, revealing a range of different mechanisms of action such as membrane pore formation, inhibition of replication, or the modulation of other immune responses [10,11,12]. When different AMPs are present at the same time, they may show additive, potentiating or synergistic effects depending on which mechanisms of action are involved, and AMPs can also show synergistic effects with conventional antibiotics [13,14]. The advantages of such effects include the lower effective dose of each compound and the reduced likelihood that pathogens will evolve resistance, given that multiple simultaneous adaptations would be necessary for different drug targets [15,16,17]. Furthermore, the structure of AMPs allows them to be designed or adjusted by adding, removing or replacing individual amino acids or modifying particular residues. This has resulted in new classes of peptidomimetic antimicrobials with improved stability and bioactivity profiles [18]. However, the delivery of multiple AMPs or AMPs combined with conventional drugs requires the synthesis and formulation of different products, which increases the costs involved and the potential for treatment failure. One way to overcome this limitation in the context of multiple AMPs is the creation of hybrid peptides in which individual AMPs are joined by linkers. A recent study showed that the hybrid peptide P7A3 was more potent than the two parental peptides, but the hemolytic activity was also much higher [19].

Here we tested the potential of two arthropod AMPs by comparing the parental AMPs to hybrids containing glycine linkers of two or six residues. The first parental candidate was CopA3, a nonomeric derivative of the longer defensin polypeptide coprisin (originally isolated from the Korean dung beetle *Copris tripartitus*) with a native histidine residue replaced with leucine to increase overall hydrophobicity [20]. CopA3 is active against the yeast *Candida albicans* [21] and the bacteria *Escherichia coli* and *Staphylococcus aureus* [20], including antibiotic-resistant strains of *E. coli*, *S. aureus* and *Pseudomonas aeruginosa* [21]. CopA3 was also cytotoxic when tested against certain cancer cells [20,21,22]. The second parental candidate was the α-helical peptide Hp1090, isolated from the venom of the Asian forest scorpion *Heterometrus petersii* [23]. Hp1090 was shown to inhibit the replication of the hepatitis C virus [23], suggesting that CopA3 and Hp1090 differ in their mechanisms of action. We investigated the role of glycine spacers on the antibacterial activity of the hybrid peptide by constructing variants separated by two (InSco2) or six (InSco6) glycine residues. We compared the antibacterial and cytotoxic activities of the parental AMPs and the hybrids InSco2 and InSco6.

## 2. Results

### 2.1. Peptide Sequences and Properties

The CopA3 and Hp1090 AMPs are nine and 13 amino acids in length, respectively (Table 1). InSco2 and InSco6 were designed based on the combination of the peptides CopA3 (in the N-terminal position) and Hp1090 (in the C-terminal position) separated by glycine linkers of two or six residues, respectively. The resulting peptides were 24 (InSco2) and 28 (InSco6) amino acids in length, respectively. The peptides were predicted to be cationic with theoretical isoelectric points of 10.48 (Table 1). InSco2 was the most hydrophobic of the peptides. Both hybrid peptides carried a slightly more positive net charge than the parental peptides.

### 2.2. Predicted 3D Structure and Membrane Polarity

**Figure 1:**
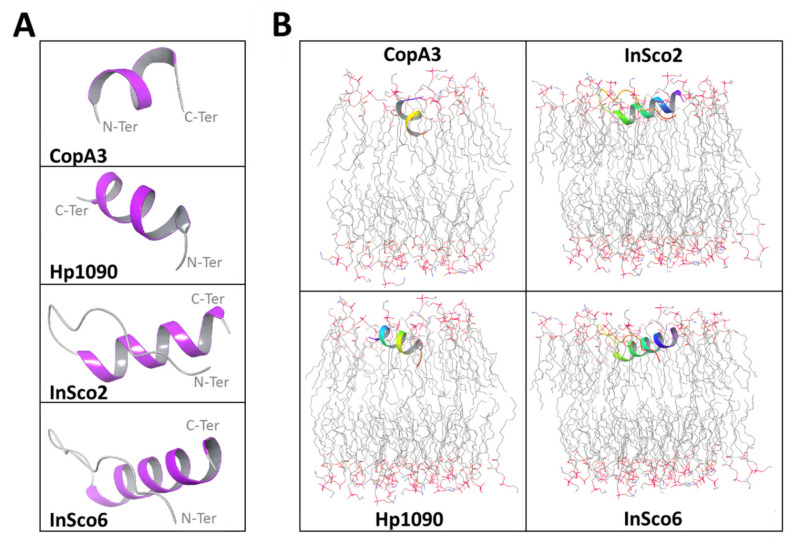
Predicted AMP structures and membrane orientations. The initial conformation of each AMP (**A**) is colored according to the secondary structure fold (gray = coil, purple = α-helix). The AMPs were oriented in a POPE bilayer membrane (**B**) with the termini color coded (red = N-terminus, purple = C-terminus). The POPE membrane atoms are also color-coded (gray = carbon, red = oxygen, blue = nitrogen; hydrogen atoms are not shown).

Initial structural analysis of the AMPs predicted α-helical folds with terminal coils (Figure 1A). The predicted α-helical fold in CopA3 is supported by the published crystal structure [24]. In the hybrid AMPs, the N-terminal coil is extended up to the glycine linker insertion (Figure 1A). Each AMP except CopA3 was predicted to align with and embed within the upper leaflet of the phosphatidylethanolamine (POPE) bilayer, whereas the orientation of CopA3 is perpendicular, approximating a right angle to the membrane (Figure 1B).

**Figure 2:**
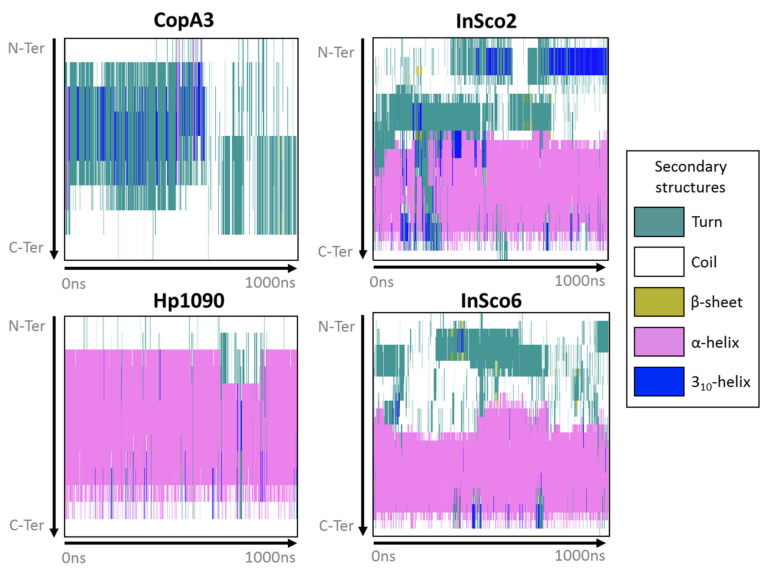
Secondary structure of the AMPs during membrane interactions. The panels display the secondary structures defined in the key for each antibacterial AMP from the N-terminus to the C terminus (*y*-axis) during the 1-µs molecular dynamics simulation (*x*-axis).

Most of Hp1090 and the C-terminus of InSco2 and InSco6 maintained an α-helical fold while interacting with the POPE bilayer during the 1-μs molecular dynamics simulation (Figure 2). However, CopA3 immediately interchanged between a turn/coil and a 3_10_-helix for ~750 ns, then converted to a turn/coil for the remainder of the simulation (Figure 2). The main difference between the hybrid AMPs was that the N-terminus of InSco2 folds into a more complex secondary structure than InSco6 (Figure 2). The N-terminus of both hybrid AMPs refolded from a coil to a turn, with the sporadic formation of β-sheets. However, the N-terminus of InSco2 folded into a 3_10_-helix for more than half of the simulation, whereas a 3_10_-helix only formed at the N-terminus of InSco6 for ~30 ns during the first half of the simulation (Figure 2).

### 2.3. Antibacterial Activity Assays

**Figure 3:**
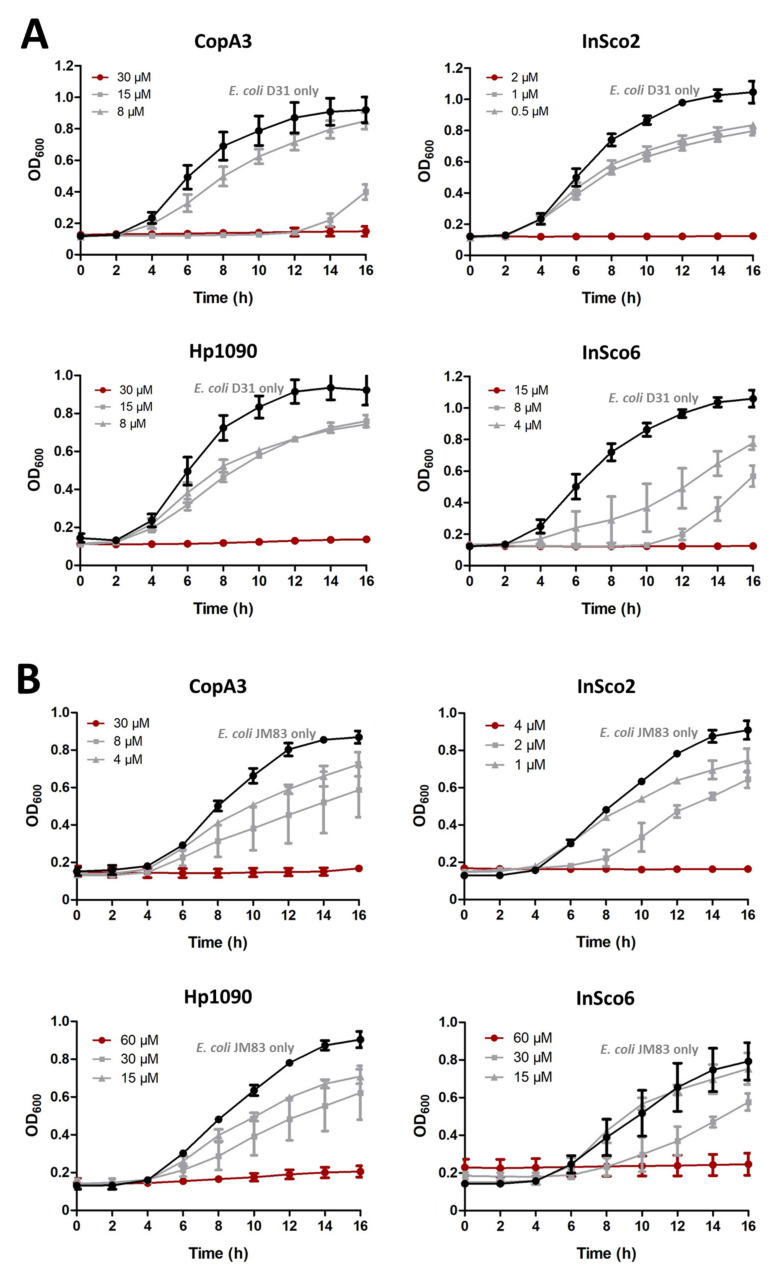
Antibacterial dose-response curves. The parental and hybrid peptides were tested against *E. coli* strains D31 (**A**) and JM83 (**B**) across the concentration range 0.015–250 µM. Concentration-response curves were generated using decreasing peptide doses. The *y*-axis shows the optical density at 600 nm (OD_600_) and the *x*-axis shows the assay time from 0 to 16 h. Each panel shows the total growth inhibition peptide concentrations (red line), different concentrations (gray lines) and the normal bacterial growth curve in the absence of AMPs (black line).

The antibacterial activity of the two parental and two-hybrid AMPs in the concentration range 0.015–250 µM was tested against *E. coli* strains D31 (Figure 3A) and JM83 (Figure 3B). All four AMPs inhibited bacterial growth; with a minimum inhibitory concentration (MIC), ranging from 2 to 60 µM. InSco2 was 15 times more potent than CopA3 and Hp1090 against *E. coli* D31, whereas InSco6 was only twice as potent. InSco2 was also 7.5 times more potent than CopA3 and 15 times more potent than Hp1090 against *E. coli* JM83, whereas InSco6 was comparable in activity to Hp1090. InSco2 demonstrated the most potent antibacterial activity against both *E. coli* strains.

### 2.4. Screening AMPs for Hemolytic and Cytotoxic Activity

**Figure 4:**
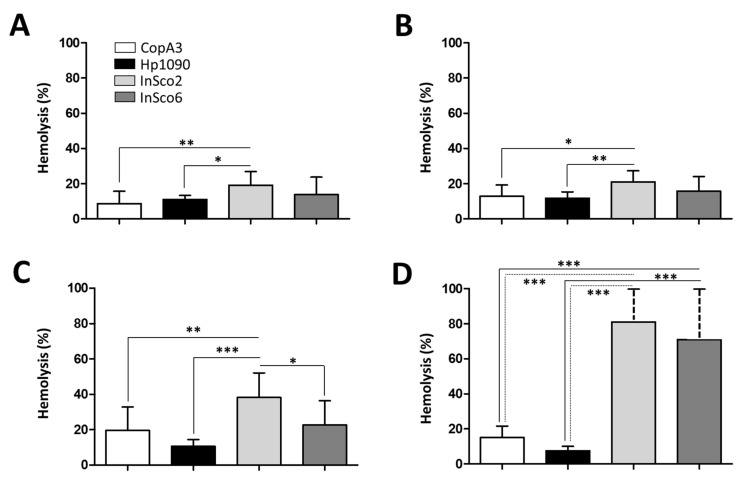
Hemolytic profile of the parental and hybrid AMPs. CD-1 mouse erythrocytes were incubated with the four AMPs to evaluate their hemolytic profiles at four concentrations: (**A**) 0.1 µM, (**B**) 1 µM, (**C**) 10 µM and (**D**) 100 µM. Results were compared by one-way ANOVA with Tukey’s multiple comparison test applied for individual comparisons (significant differences between AMPs are indicated as follows: * *p* < 0.05; ** *p* <0.001; *** *p* < 0.0001).

As an indicator of potential toxicity, we next tested the hemolytic activity of the two parental and two-hybrid peptides, each at four different concentrations (0.1, 1, 10 and 100 µM) (Figure 4A–D). At all four concentrations, InSco2 showed significantly greater hemolytic activity than both parental peptides (Figure 4) and at a concentration of 10 µM, the hemolytic activity of InSco2 was also greater than that of InSco6 (Figure 4C). At the highest concentration of 100 µM, both InSco2 and InSco6 showed significantly greater hemolytic activity than the parental AMPs (Figure 4D). The potent antibacterial activity of InSco2 in particular therefore appears to present an undesirable collateral risk of toxicity.

**Figure 5:**
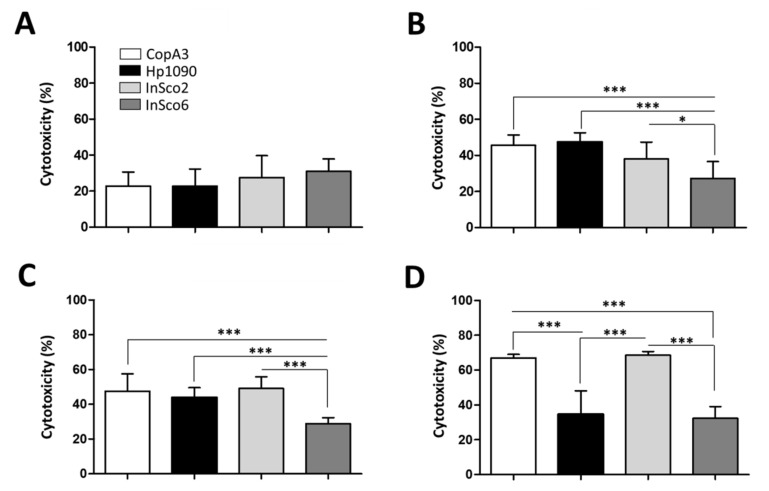
Cytotoxicity profile of the parental and hybrid AMPs. BHK-21 cells were incubated with the four AMPs to evaluate their cytotoxicity at four concentrations: (**A**) 0.1 µM, (**B**) 1 µM, (**C**) 10 µM and (**D**) 100 µM. Results were compared by one-way ANOVA with Tukey’s multiple comparison test applied for individual comparisons (significant differences between AMPs are indicated as follows: * *p* < 0.05; *** *p* < 0.0001).

The cytotoxicity of the AMPs was also tested against BHK-21 cells at four concentrations (0.1, 1, 10 and 100 µM) (Figure 5). At the lowest peptide concentration (0.1 µM) no significant differences in cytotoxicity were detected (Figure 5A), but significant differences were evident at higher concentrations (Figure 5B–D). CopA3 and InSco2 showed a dose-dependent increase of cytotoxicity, reaching almost 70% at the highest concentration (Figure 5D). Hp1090 reached maximum cytotoxicity (~45%) at 1 µM (Figure 5B) and the cytotoxicity of InSco6 remained at ~30% regardless of the concentration.

### 2.5. Synergistic Activity of CopA3 and Hp1090

**Figure 6:**
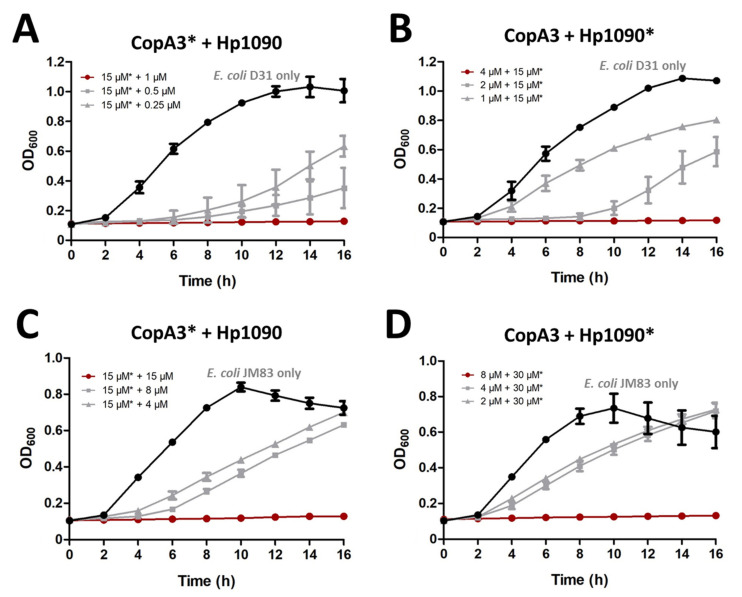
The combined effect of CopA3 and Hp1090 against two *E. coli* strains. The combined antibacterial activity of the parental AMPs was tested against *E. coli* D31 (**A**,**B**) and *E. coli* JM83 (**C**,**D**). (**A**,**C**) The sublethal concentration of CopA3 was set at 15 µM and was combined with increasing concentrations of Hp1090. (**B**) The sublethal concentration of Hp1090 was set at 15 µM and was combined with increasing concentrations of CopA3. (**D**) The sublethal concentration of Hp1090 was set at 30 µM and was combined with increasing concentrations of CopA3. The AMP with the constant concentration is marked with an asterisk. The OD_600_ was recorded using a microtiter plate reader. In each panel, the red line indicates the total inhibition of the combined treatment, the gray line shows the decreasing concentration of the serially-diluted AMP with the other sublethal concentration and the black line shows the bacterial growth curve in the absence of AMPs.

For the *E. coli* strain D31, sublethal concentrations of CopA3 (15 μM) were combined with serial dilutions of Hp1090 and vice versa to investigate the potential for synergistic activity between the parental peptides. Sublethal concentrations of CopA3 with 1 µM Hp1090 completely inhibited the growth of *E. coli* D31 (Figure 6A), but sublethal concentrations Hp1090 required 4 µM of CopA3 to achieve the same effect (Figure 6B). For the *E. coli* strain JM83, sublethal concentrations of CopA3 (15 µM) were combined with serial dilutions of Hp1090, and sublethal concentrations of Hp1090 (30 µM) were combined with serial dilutions of CopA3. For the first combination (15 µM CopA3 and serial dilution of Hp1090), 15 µM of Hp1090 was required for the total inhibition of *E. coli* JM83 (Figure 6C). For the second combination (30 µM Hp1090 and serial dilution of CopA3), 8 µM of CopA3 was required for the total inhibition of *E. coli* JM83 (Figure 6D).

To confirm the existence of a synergistic effect in the activity between the parental peptides, we calculated the fractional inhibitory concentration index (FIC_index_) for both strains of *E. coli*. FIC_index_ values below 0.5 are considered synergistic and those between 0.5 and 1 are considered partially synergistic [25]. The combined treatments with CopA3 and Hp1090 were synergistic against *E. coli* D31 (FIC_index_ = 0.17) and also partially synergistic against *E. coli* JM83 (FIC_index_ = 0.52) (Table 2).

## 3. Discussion

The widespread and indiscriminate use of conventional antibiotics has led to the emergence and spread of MDR bacteria, including pathogens that are resistant to antibiotics of last resort. The development of new antimicrobial candidates with potent activity and novel mechanisms of action is, therefore, a high priority [26]. AMPs fulfil these requirements, targeting a broad range of pathogens at low concentrations, with little evidence of resistance thus far [19,27]. However, AMPs will only be suitable for clinical development if we can overcome challenges such as cytotoxicity, immunogenicity, and loss of activity in vivo [28]. Strategies to facilitate the development of AMPs include biochemical modifications and peptide engineering approaches, including the creation of hybrid peptides [29,30].

In this study, we investigated the individual and combined activity of two arthropod AMPs selected based on their presumed distinct mechanisms of action, making it more likely they will demonstrate synergistic activity. As well as testing the AMPs individually and in combination, we also created hybrid peptides in which the parental sequences were separated by two or six glycine residues. Glycine linkers are often used in fusion proteins, and the length of the linker influences the conformational freedom of the fusion partners and thus the efficiency of target interactions [31]. Glycine spacers also play a key role in the structural stability of protein scaffolds and can improve the effectiveness of functional peptides [32]. For example, the introduction of glycine into the AMP kiadin improved its antimicrobial activity [31] while modulating the structural and functional dynamics of self-assembled peptides [33]. However, to the best of our knowledge, this is the first report in which glycine spacers have been used as the hinge region between two distinct AMPs.

Gram-negative bacteria are particularly challenging as pathogens because they feature a peptidoglycan layer with an additional lipopolysaccharide outer membrane, which confers protection against many conventional treatments [34]. The inner cytoplasmic membrane of Gram-negative bacteria consists of a mixture of zwitterionic and anionic phospholipids such as POPE [34], and includes many negatively charged phosphate groups resulting in low permeability and the exclusion of most hydrophobic AMPs [35]. The activity of many AMPs involves the permeabilization of bacterial membranes, inducing cell lysis [36,37]. AMPs that act against Gram-negative bacteria must therefore disrupt both the inner and outer membranes [37].

In our study, we predicted that three of the four AMPs would initially embed in the upper leaflet of the POPE bilayer in parallel alignment, whereas CopA3 penetrated into the membrane in a perpendicular orientation. This is consistent with two distinct mechanisms: one involving direct pore formation and the other based on the so-called carpet model. At low concentrations, many AMPs align parallel to the membrane either as monomers or aggregates. If the concentration increases above a certain peptide-to-lipid ratio, some AMPs can reorient perpendicular to the membrane, allowing them to form barrel stave or toroidal pores [38,39,40]. In contrast, the carpet model is adopted by AMPs that lack a specific pore-forming capacity [41,42]. Here, the AMPs align parallel to the lipid bilayer and accumulate until they cover the membrane surface, resulting in a detergent-like effect that causes membrane disintegration [43] and ultimately membrane depolarization and cell death [41,44,45]. Our predicted membrane interactions suggest that CopA3 works by directly generating pores in the membrane, as previously reported for magainin 2 [46] and melittin [44,45,46], whereas Hp1090, InSco2 and InSco6 use the carpet model, as previously reported for cecropin [43]. However, the activity of AMPs also depends on the composition of the lipid bilayer [47,48]. For example, auerin 2.2 forms toroidal pores in a model membrane comprising a 1:1 mixture of 1-palmitoyl-2-oleoyl-*sn*-glycerol-3-phosphocholine (POPC) and 1-palmitoyl-2-oleoyl-*sn*-glycero-3-phospho-(1′-*rac*-glycerol) (POPG) but uses the carpet model when the membrane comprises a 1:1 mixture of 1,2-dimyristoyl-*sn*-glycero-3-phosphocholine (DMPC) and 1,2-dimyristoyl-*sn*-glycero-3-phospho-(1′-rac-glycerol) (DMPG) [47,48].

The antimicrobial activity, toxicity and selectivity of AMPs are related to multiple physicochemical properties including peptide length, charge, hydrophobicity and amphipathicity [19,49,50,51]. For example, the α-helical analogs of a cecropin-melittin hybrid revealed that peptides with similar secondary structures, and minimal differences in primary sequence, can nevertheless confer different antibacterial activities [4,52]. We found that both hybrid AMPs were more potent than the parental CopA3 and Hp1090 peptides, especially InSco2. Short peptides containing 2-aminoisobutyric acid (AIB) tend to assume 3_10_-helical conformations [53] and AIB substitutions in the frog skin peptide temporin-1DRa increased its antimicrobial and cytolytic activities [54]. The N-terminus of InSco2 forms a 3_10_-helix whereas the N-terminus of InSco6 remains in a coil configuration. InSco2 may therefore incorporate AIB, leading to the formation of a 3_10_-helix and thus possibly explaining the potent activity of InSco2 against bacteria (as well as the stronger hemolytic and cytotoxic activities compared to InSco6 and the parental AMPs).

Positively charged AMPs bind to negatively charged phospholipids on the outer leaflet of the bacterial membrane via electrostatic interactions [41]. Increasing the positive charge should therefore enhance such interactions and confer greater activity [55,56]. However, whereas increasing the charge on the AMP magainin from +3 to +5 was shown to increase its activity against both Gram-positive and Gram-negative bacteria due to increased affinity for the bacterial membrane, a further increase to +6 or +7 caused the antibacterial activity to decline while increasing hemolytic activity [55]. The loss of activity following an increase in charge suggests a more complex interaction [55]. CopA3 and Hp1090 have net charges of +2 and +3, respectively, while both hybrid peptides have a charge of +5. The higher net charge on the hybrid peptides correlates with their higher antibacterial activities, although a direct causative link would require more detailed analysis (and other factors are likely to be involved given the significant difference in activity between InSco6 and InSco2).

In theory, antibacterial activity can be decoupled from hemolysis because bacterial membranes feature anionic lipids whereas the membranes of erythrocytes are neutral [30]. This should allow the development of potent but safe AMP-based therapeutics [57]. The biological activity of AMPs may depend in part on their amphipathicity, although this property may be restricted to cationic AMPs that fold into an α-helix [58,59]. Generally, the amphipathicity of an AMP correlates with both its antibacterial and hemolytic activity [39]. Hydrophobicity also contributes to the ability of AMPs to interact with different membranes as well as peptide partitioning within the lipid bilayer [58,60,61]. Differences in hydrophobicity between the AMP Pin2 and its variant Pin2[G] were shown to contribute to the greater hemolytic activity of the variant [62], suggesting that optimal hydrophobicity is essential for AMP function [41]. Excess hydrophobicity has been shown to confer cytotoxicity and reduce antimicrobial selectivity [42,63]. Increasing the hydrophobicity of the non-polar face of the amphipathic α-helix also enhances hemolysis because the more hydrophobic peptides penetrate more deeply into the hydrophobic core of the erythrocyte membrane, a phenomenon known as membrane discrimination [64]. In contrast, cathelicidin and aurein derivatives with optimal amphipathicity and greater hydrophobicity than their parental peptides achieved higher antimicrobial activity and selectivity [19]. We found that the greater hydrophobicity of the hybrid peptides, especially InSco2, increased their hemolytic and cytotoxic activity compared to the parental AMPs, equivalent to a loss of selectivity. However, given the difference between the two bacterial strains, a degree of selectivity clearly remained.

Understanding the mode of action, selectivity and toxicity of AMPs will facilitate their rational design as therapeutic agents. Although AMPs show some degree of selectivity between bacteria and mammalian cells, the lethal dose in mammalian cells generally does not differ significantly from the lethal dose in bacteria [31]. However, InSco2 showed antibacterial activity at low concentrations against *E. coli* D31 (2 µM) and *E coli* JM83 (4 µM) whereas much higher concentrations (100 µM) were required for hemolytic and cytotoxic activity.

The synergy between AMPs is a particularly interesting phenomenon [14]. Mixture assays demonstrated the synergistic effect of the parental peptide combination CopA3/Hp1090 against *E. coli* strains, resulting in much higher antimicrobial activity than the individual peptides. Based on the proposed mode of action of the parental peptides, we assume that Hp1090 has the ability to disrupt the *E. coli* outer membrane by lying parallel on its surface, promoting the uptake of CopA3 into the cells. Hp1090 appears to potentiate the activity of CopA3 by making the membrane more permeable, allowing the latter to gain access to its intracellular targets. This enhanced synergistic activity would involve both peptides acting initially in the same location, explaining why the mode of action is preserved in the hybrid peptides.

## 4. Materials and Methods

### 4.1. Peptide Sequences and Bioinformatics

The CopA3 and Hp1090 peptides [20,23] were prepared by solid-phase synthesis and the crude products were purified by reversed-phase high-performance liquid chromatography (RP-HPLC) on a Venusil XBP-C18 4.6 × 250 mm column. The purity of the resulting peptides was >85% (Covalab, France). They were lyophilized and stored at −20 °C. The molecular weight and isoelectric point of each peptide were predicted using the ExPASy tools server [65,66,67]. Hydrophobicity was calculated using the peptide analysis tool (Thermo Fisher Scientific, USA) and the net charge at pH 7 was determined using the Bachem peptide calculator.

### 4.2. In silico Peptide Structure Prediction

The sequences of Hp1090 [23], InSco2 and InSco6 were uploaded to the I-TASSER protein structure prediction server [68]. The CopA3 sequence [24] was generated by truncating the coprisin peptide from the Protein Databank (PDB) structure 2LN4 [24] and introducing the mutation His23Leu to generate the coprisin analog CopA3 [20]. The AMP structures were prepared separately and their hydrogen-bond networks were optimized using the Protein Preparation Wizard [69] followed by global minimization using default settings to remove any steric clashes [65,66,70].

### 4.3. Peptide-Membrane Molecular Dynamics

The optimized AMP structures were uploaded to the PPM server [71] to determine membrane orientation, providing templates to build a POPE bilayer membrane using Desmond [72] in an orthorhombic box (buffer 10 Å^3^) with a TIP3P explicit model, neutralized and salted with 0.15 M NaCl. The following force fields were used to parameterize the AMP-membrane systems: TIP3P CHARMM [73] for the solvent, AMBER99SB-ILDN [74,75,76] for the AMPs, and CHARMM36 [77] for the POPE membrane and ions. Molecular dynamics were then carried out in Desmond (semi-isotropic conditions) for 1 μs using an NPγT ensemble coupled with a Nose-Hoover thermostat [78] and a Martyna-Tobias-Klein barostat [79]. The surface tension was set to 4000 bar and the temperature to 310 K with a RESPA [80] integrator at an inner time step of 2.0 fs. All calculations were conducted using a GPU-accelerated workstation and analyzed using the Maestro software package (Schrödinger, USA) and Visual Molecular Dynamics [81].

### 4.4. Minimum Inhibitory Concentration

The minimum inhibitory concentration (MIC) of the AMPs was determined using the *E. coli* strains D31 and JM83. Each strain was cultured in a Falcon tube overnight at 37 °C in lysogeny broth (LB) liquid medium (Sigma-Aldrich, Germany). The suspensions were then diluted in LB medium to reach an optical density at 600 nm (OD_600_) of 0.001. An AMP stock solution of 1 M was prepared by dissolving the AMPs completely in double-distilled water. Serial dilutions of each AMP (0.015, 0.03, 0.06, 0.12, 0.25, 0.5, 1, 2, 4, 8, 15, 30, 60, 120 and 250 µM) were then used to determine antibacterial activity as previously reported [82]. Negative control cultures without AMPs were also included. The MIC was defined as the lowest AMP concentration causing the total inhibition of bacterial growth. The assays were carried out at least three times with comparable results.

### 4.5. Measurement of Hemolytic Activity and Cytotoxicity

The hemolytic activity of the AMPs was tested against CD-1 mouse blood mixed with the anticoagulant dipotassium EDTA (Dunn Labortechnik, Germany) as previously described [83]. Briefly, erythrocytes were harvested by centrifugation at room temperature and washed three times with phosphate-buffered saline (PBS). A 1:20 suspension of erythrocytes in PBS was transferred to 96-well plates and incubated for 1 h with diluted AMPs (0.1, 1, 10 and 100 µM). The cells were then centrifuged, and the supernatant was transferred to a fresh plate for absorbance measurement at 570 nm (A_570_ nm) using an Eon microplate spectrophotometer (BioTek Instruments, USA).

The cytotoxicity of the AMPs was evaluated using baby hamster kidney fibroblasts (BHK-21 cell line) included in the F2H Kit Basic (Chromotek, Germany). The cells were grown in Dulbecco’s modified Eagle’s medium (DMEM) supplemented with 4.5 g/L glucose, 110 mg/L sodium pyruvate, 584 mg/L l-glutamine and 10% fetal bovine serum (FBS). The cells were maintained in a NU-5810 incubator (IBS Tecnomara, Germany) at 37 °C with a 5% CO_2_ atmosphere. The cells were then sub-cultured at 85–90% confluence by detaching with 0.25% trypsin and 0.03% EDTA (Sigma-Aldrich). The AMP stocks in water (see above) were then diluted in DMEM to final concentrations of 0.1, 1, 10 and 100 µM. One day before each experiment, cells were transferred to Greiner Cell Star 96-well culture plates (Sigma-Aldrich) at a density of 1 × 10^5^ cell/mL. The cells were rinsed with 100 µL PBS before adding 100 µL of each AMP dilution and then incubated for 3 h at 37 °C for 1.5 h. The AMPs were then removed by washing the cells three times with PBS. Cell viability was determined using 10% (*v*/*v*) AlamarBlue reagent (Bio-Rad Laboratories, Germany) in DMEM. Fluorescence was measured in an Eon microplate reader (excitation = 528 nm, emission = 590 nm). Cells exposed to DMEM without AMPs were used as a negative control, and DMEM only was used as the blank reference. Three independent experiments were carried out in triplicate for each AMP concentration.

### 4.6. Synergistic Effect of CopA3 and Hp1090

Sublethal concentrations of CopA3 and Hp1090 were used in mixture assays against *E. coli* strains D31 and JM83 to assess their synergistic effect. We used the half dose lethal concentrations (LD_50_), which do not cause growth inhibition [84]. The sublethal concentration of CopA3 was set at 15 µM with serial dilutions of Hp1090. The mixture assay was repeated in reverse with the sublethal concentration of Hp1090 (15 or 30 µM, depending on the *E. coli* strain) and serial dilutions of CopA3. In both cases, the MIC of CopA3 and Hp1090 from these combinations was then recorded. Based on the results, fractional inhibitory concentration indices (FIC_index_) were calculated to determine any interactions between the parental AMPs. The ΣFIC_index_ is a combination of FIC indices from both parental peptides and was calculated using the following equation:

ΣFIC_index_ = FIC_CopA3_ + FIC_Hp1090_ = (C_CopA3_/MIC_CopA3_ + C_Hp1090_/MIC_Hp1090_)

where MIC_CopA3_ and MIC _Hp1090_ are the MIC of individual parental peptides, and C_CopA3_ and C_Hp1090_ are the MIC of the parental peptides in combination [25].

### 4.7. Statistical Analysis

For hemolysis and cytotoxicity, statistical differences between AMPs were evaluated by one-way analysis of variance (ANOVA) with Tukey’s multiple comparison test applied for individual comparisons in GraphPad Prism v5 (GraphPad Software, USA). Differences were considered significant at a threshold of *p* < 0.05.

## 5. Conclusions

To our knowledge, this is the first report that combines insect and chelicerate AMPs with glycine spacers to create hybrid peptides that improve antimicrobial activity. We conclude that a glycine spacer of at least two residues can improve the activity of AMPs without affecting their selectivity. We propose that the ability of the extended N-terminal coil of InSco2 to fold into a 3_10_-helix may enhance its antibacterial properties. The rational design of hybrid AMPs could therefore be improved by considering the temporal folding of the terminal regions of each peptide in relation to their targets in the cell membrane. These rational modifications will optimize the selectivity of AMPs and improve their therapeutic indices as novel antimicrobial agents.

## Figures and Tables

**Table 1:** Sequence information of the parental and hybrid AMPs and predicted physicochemical properties.

Peptides	Sequence	MW	pI	H	Net Charge *
*Parental*					
CopA3	LLCIALRKK	1.05	10.06	26.41	+3
Hp1090	IFKAIWSGIKSLF	1.5	10.00	44.75	+2
*Hybrid*					
InSco2	LLCIALRKKGGIFKAIWSGIKSLF	2.66	10.48	54.64	+5
InSco6	LLCIALRKKGGGGGGIFKAIWSGIKSLF	2.89	10.48	50.18	+5

MW = molecular weight; pI = isoelectric point; H = hydrophobicity. * Net charge at pH 7.0.

**Table 2:** The fractional inhibitory concentration index (FIC_index_) of the combined treatment with parental AMPs CopA3 and Hp1090 against *E. coli* strains D31 and JM83.

Bacteria	Peptides	C_alone_ (µM)	MIC_c_ (µM)	FIC_CopA3_ + FIC_Hp1090_	Combination Effect
*E. coli* D31	CopA3	30	4	0.17	Synergy
	Hp1090	30	1		
*E. coli* JM83	CopA3	30	8	0.52	Partial synergy
	Hp1090	60	15		

MIC_C_ = minimum inhibitory concentration, in combination; FIC_index_ = fractional inhibitory concentration index.

## Data Availability

Not applicable.
